# Piezo1 in Peripheral and Central Sensitisation: Implications for Chronic Pain

**DOI:** 10.3390/bioengineering13091074

**Published:** 2026-09-16

**Authors:** Olga Kopach

**Affiliations:** 1 School of Health & Medical Sciences, City St. George’s University of London, London SW17 0RE, UK; okopach@sgul.ac.uk or o.kopach@ucl.ac.uk; 2 Research Department of Epilepsy, Queen Square Institute of Neurology, University College London, London WC1N 3BG, UK

**Keywords:** Piezo1, mechanobiology of chronic pain, nociceptive pathways, central sensitisation in the spinal cord, maladaptive plasticity, therapeutic strategies

## Abstract

Piezo1 is an increasingly recognised mechanosensitive ion channel that transduces mechanical forces into electrical and biochemical signals and is expressed in peripheral sensory pathways as well as across multiple cell types within the central nervous system. Growing evidence indicates that Piezo1 contributes to nociceptive processing by influencing cellular excitability, inflammatory signalling, and neuron–glia interactions within nociceptive circuits. These actions position Piezo1 as an important contributor to amplified nociceptive transmission and circuit hyperexcitability, thereby potentially facilitating the transition from acute to chronic pain via several integrating mechanisms. In this context, Piezo1 may interact with other established mechanisms of chronic pain, including peripheral and central sensitisation and neuroinflammatory signalling, to help sustain persistent pain states. This review examines the potential roles of Piezo1 as a synergistic contributor to nociceptive hyperexcitability and considers how it may modulate nociceptive excitability, glial activation, and central sensitisation. Available findings support the view that Piezo1 participates in mechanotransduction and maladaptive plasticity relevant to chronic pain, although direct evidence for its cell-specific actions within spinal dorsal horn circuits remains limited. Piezo1 has also been implicated in tissue remodelling following spinal cord injury, suggesting that its therapeutic relevance may extend beyond pain signalling alone. Further studies will be required to clarify its mechanistic role in the mechanobiology of chronic pain and to evaluate the translational potential of targeting Piezo1 through pharmacological or genetic strategies for pain management.

## 1. Introduction

Chronic pain remains among the most challenging clinical conditions to treat, in part because its pathophysiology extends beyond the initiating tissue injury and involves persistent changes in neural signalling, circuit excitability, and neuroimmune interactions. Among the cellular contributors increasingly implicated in nociception are mechanosensitive ion channels, which have emerged as significant drivers of hyperexcitability and nociceptive amplification, highlighting a potential role for mechanobiology in the persistence of pain [1,2].

The mechanically activated Piezo ion channel family in mammals comprises the Piezo1 channel, encoded by the *Piezo1* gene (formerly designated *FAM38A*) and the Piezo2 channel, encoded by *Piezo2* (formerly *FAM38B*). These channels enable cells to convert physical forces into electrical and biochemical signals. The PIEZO1 and PIEZO2 proteins are unique in their large size and share ∼42% amino acid identity, while lacking homology to other membrane proteins. In peripheral sensory neurons, mechanically evoked touch and proprioception are primarily mediated by Piezo2 [3,4], which explains why Piezo1 has historically attracted less attention in studies of sensory signal transduction. Nevertheless, emerging evidence indicates that Piezo1 contributes more broadly to sensory processing within both peripheral and central nervous systems (CNS) than previously thought, encompassing touch perception, proprioception, mechanical itch [5], pain-related signalling in migraine [6], and neuropathic pain [7].

Piezo1 was first identified in 2010 as a mechanically activated, non-selective cation channel [8], an advance that marked a significant milestone in the field of mechanobiology by establishing a distinct class of force sensor. Structurally, Piezo1 assembles into a trimeric membrane complex possessing a propeller-like architecture that responds to membrane tension and the lipid bilayer perturbation; the conformational changes accompanying this response permit a relatively modest opening of a central pore, allowing cation permeation [9,10]. The human Piezo1 central pore conducts monovalent ions, including Na^+^, K^+^, and Cs^+^, together with divalent cations such as Ca^2+^, Mg^2+^, Ba^2+^, and Mn^2+^, and some organic cations, including tetramethylammonium and tetraethylammonium [11]. Although the channel has limited ion selectivity, its robust Ca^2+^ permeability is of particular functional importance, since Piezo1-driven Ca^2+^ influx couples mechanical stimuli to intracellular signalling pathways that govern cellular excitability, gene expression, and longer-term cellular responses to adaptive changes in cellular function.

The characteristically slower gating kinetics of Piezo1, compared with its homologue Piezo2 [12], together with its relatively slow inactivation [13,14], favour more sustained Piezo1 activation under conditions of prolonged mechanical stress. Inactivation, as one of the key regulatory mechanisms governing Piezo channel function, determines the probability of channel opening during sustained mechanical stimulation, and multiple Piezo1 mutations—at least 25 of which have been currently characterised—are known to alter channel gating kinetics associated with human diseases [15], including mutations linked to spinal degeneration [16]. Moreover, Piezo1 is notably dynamic within the plasma membrane, diffusing freely and preferentially localising to sites of elevated cellular tension while interacting with matrix adhesions [17,18]. The fact that Piezo1 is not static within the plasma membrane indicates that Piezo1 is capable of tuning cellular responses to a range of physical forces rather than functioning as a fixed, static sensor. In addition, Piezo1 can interact with the sarcoplasmic/endoplasmic reticulum Ca^2+^ ATPase (SERCA) via a direct protein–protein interaction, which can modulate its mechanogating properties [19]. Taken together, these structural and biophysical properties provide a mechanistic basis for considering Piezo1 as a sustained regulator of cellular excitability, functioning in both neuronal and non-neural cell types, under physiological and pathological conditions, rather than a transient mechanical sensor alone.

This review examines the mechanobiology of sustained hypersensitivity within nociceptive circuits, with a particular focus on Piezo1 as a cellular contributor to peripheral and central sensitisation, extending its potential role in nociplastic hypersensitivity. It first outlines the distribution of Piezo1 across neural and non-neural cell types implicated in nociceptive processing, then considers how Piezo1 may influence synaptic transmission and neuroinflammatory signalling, and finally discusses how this knowledge might be translated into strategies for targeting Piezo1 in chronic pain management.

## 2. Cell-Type-Specific PIEZO1 Expression and Distribution in Nociceptive Pathways

Within the nervous system, Piezo1 is expressed across a wide range of cell types and anatomical compartments, including neurons [20], astrocytes [21,22], microglia, satellite glia [23,24], Schwann cells [25], endothelial cells [26,27], brain-derived neural stem/progenitor cells [28], and erythroid progenitor cells [29]. This widespread distribution suggests that Piezo1 functions as a broadly deployed mechanosensitive component of neural tissue, rather than being confined to a single, specialised cell population.

Transcriptomic and protein-level studies indicate that PIEZO1 expression is highly cell-type- and region-specific rather than uniform, even within the same anatomical compartment. In the peripheral sensory system, Piezo1 is expressed in primary sensory neurons, including mouse and human dorsal root ganglia (DRG) neurons [12,30,31], with approximately 25% of DRG neurons reported to express Piezo1 [32], as well as in non-neuronal cells of the DRG [23], trigeminal ganglion neurons [33,34], and in the nodose ganglion neurons [35]. Several studies report enrichment of Piezo1 in small- and medium-diameter DRG neurons, particularly in TRPV1-positive nociceptors [32], and PIEZO1 transcripts are likewise preferentially enriched in smaller-diameter DRG neurons [36]. PIEZO1 has also been detected at the central presynaptic terminals of nociceptive DRG axons [30] that converge within the dorsal horn (DH) of the spinal cord onto second-order spinal neurons, thereby placing Piezo1 in a strategically advantageous position to influence neurotransmitter release at the first central synapse. This localisation provides an anatomical basis for the hypothesis that Piezo1 could couple peripheral mechanical signals to altered synaptic drive within DH circuits, where nociceptive inputs are integrated before ascending to higher sensory centres. Under conditions of prolonged peripheral nociceptive drive, this would plausibly amplify synaptic transmission and promote postsynaptic plasticity at the level of central nociceptive circuits.

Piezo1 is not restricted to peripheral neurons. The channel is also expressed in DRG satellite glia, astrocytes, microglia, and Schwann cells [24,25], as well as in non-neural subtypes, such as vascular endothelial cells [26], smooth muscle cells of small-diameter arteries [37], infiltrating macrophages [38], and blood cells [39]. This expression pattern is notable given that satellite glia regulate extracellular ionic balance, provide trophic support, and modulate inflammatory signalling within the local microenvironment, all of which may influence nociceptor excitability. PIEZO1 is also highly expressed in epidermal keratinocytes, non-neuronal cutaneous cells that are increasingly recognised as important regulators of normal sensory afferent firing and touch sensation [40]. Collectively, these findings indicate that Piezo1-dependent modulation of pain signalling is unlikely to be neuron-specific and instead likely reflects coordinated activity across both neural and non-neural cell populations.

In the CNS, PIEZO1 has been detected across multiple regions, although its expression varies considerably according to anatomical site and cell population. Higher expression levels are often associated with stronger mechanically evoked Ca^2+^ responses and depolarisation, supporting the idea that Piezo1 contributes to region-specific mechanotransduction rather than acting as an ubiquitously distributed mechanical sensor of identical function across circuits. Data from the Human Protein Atlas indicate detectable PIEZO1 expression within the brain (https://www.proteinatlas.org/ENSG00000103335-PIEZO1/brain accessed on 5 May 2026), consistent with a broad yet non-homogeneous central distribution. Within the spinal cord parenchyma specifically, Piezo1 is expressed and functionally active in astrocytes, microglia, and macrophages [41], where mechanical stress can trigger Piezo1-mediated Ca^2+^ influx and downstream pro-inflammatory responses. Such cellular diversity suggests that Piezo1 may influence nociceptive processing across several distinct levels, spanning primary afferent excitability, spinal integration, and neuroimmune modulation.

In neurons, Piezo1 modulates membrane excitability, whereas in glial cells it may contribute to inflammatory signalling and circuit remodelling. Under physiological conditions, these actions help sustain normal network function and circuitry stability; under pathological conditions, however, excessive or dysregulated Piezo1 activity may amplify excitability, promote neuroinflammation, and facilitate maladaptive circuit plasticity. This dual functional profile is particularly notable within nociceptive circuits, where sensory DRG afferents relay nociceptive input to the spinal DH for integration and processing of nociceptive information prior to ascending toward higher centres.

Overall, the heterogeneity in Piezo1 expression across nociceptive circuits likely determines how distinct cell populations respond to mechanical stress, inflammation, or injury. This diversity further suggests that Piezo1-mediated mechanosensitivity is locally tuned according to cell type (neural or non-neural), anatomical location, and pathological context. Rather than acting merely as a mechanical sensor, Piezo1 appears to participate within a broader signalling network that integrates mechanical input, neuronal excitability, and neuroimmune activation—processes of direct relevance to the transition from acute nociceptive input to chronic pain states.

## 3. Piezo1 in Peripheral Nociceptor Excitability and Sensitisation

Piezo1 functions as a critical mechanotransducer within nociceptive pathways, converting physical stress and inflammatory signals into hyperexcitable nociceptive inputs that can persist and contribute to mechanical allodynia and hyperalgesia. Its influence extends across peripheral nerve endings, DRG neurons, and DH circuitry, indicating a role in pain processing at both primary afferents and central circuits, driving peripheral mechanosensitivity while simultaneously strengthening nociceptive inputs to central pathways and contributing to central sensitisation.

### 3.1. Piezo1-Dependent Amplification of Peripheral Nociceptive Signalling

In peripheral sensory neurons, normal touch and proprioception are primarily mediated by the mechanically gated Piezo2 channel [3,4]. Although in situ hybridisation and quantitative PCR analyses in DRG neurons demonstrate that baseline Piezo1 expression is much lower than Piezo2 [3,8], ectopic expression of Piezo1 within IB4-positive nociceptors markedly sensitised touch responses in otherwise normal mice [31]. Functional Piezo1 has additionally been identified in a subpopulation of DRG neurons that mediate mechanical itch [5]. This experimental evidence supports a direct contribution of Piezo1 to mechanical pain signalling and nociception.

**Figure 1:**
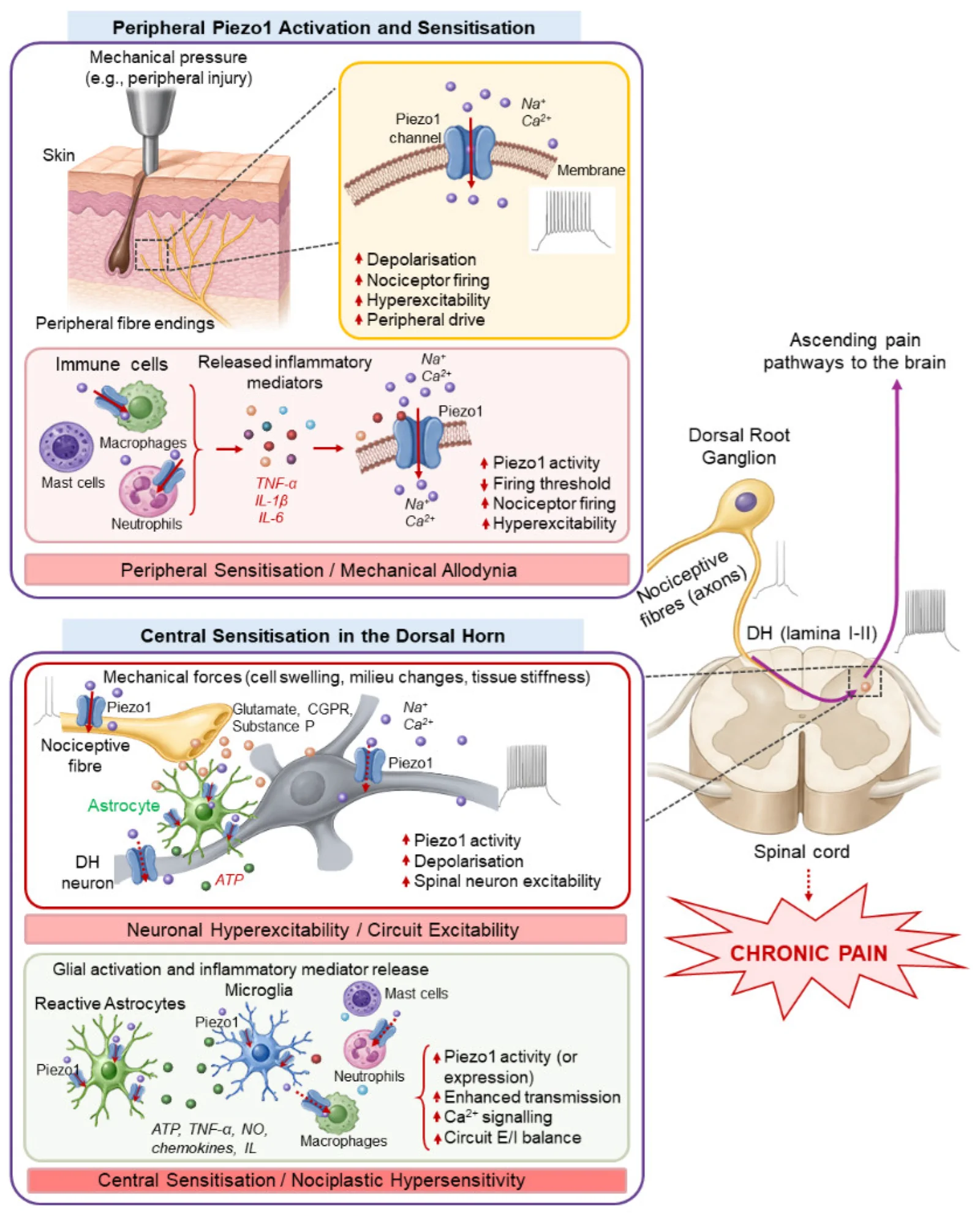
Proposed roles of Piezo1 in peripheral and central sensitisation. Schematic depicting the hypothesised contribution of Piezo1 to peripheral (**upper** panel) and central (**lower** panel) sensitisation, engaging both neuronal and glial activation to amplify nociceptive signalling directly, through membrane depolarisation, or indirectly, through local inflammatory reinforcement, ultimately contributing to nociplastic hypersensitivity and chronic pain. Solid arrows indicate pathways supported by direct experimental evidence; dashed arrows indicate proposed or hypothetical links inferred from indirect or non-spinal evidence. (Images created with BioRender, modified and assembled using Adobe Photoshop v.20 and CorelDRAWX5).

Mechanistically, Piezo1 activation enhances mechanically induced excitability by generating a transmembrane ion current associated with Ca^2+^ influx—these depolarise the membrane and lower or sensitise the threshold for action potential generation, increasing the firing probability of nociceptors [31]. Increased nociceptive excitability is, in turn, linked to increased release of pro-inflammatory and pro-nociceptive neuropeptides, such as substance P and calcitonin gene-related peptide (CGRP), which can trigger local inflammation and reinforce inflammatory signalling. During inflammation, mediators such as interleukin-1α and β (IL-1α, IL-1β), IL-6, and TNF-α further lower the activation threshold of Piezo1 [22,42,43]. Consequently, stimuli that would normally be innocuous can evoke an exaggerated influx of Ca^2+^ and Na^+^ through Piezo1, driving nociceptor firing and signal transmission to the spinal cord, which may in turn promote central sensitisation and hypersensitisation (Figure 1). Piezo1 activation may thus amplify nociceptive signalling through two complimentary routes: directly, via membrane depolarisation, and indirectly, via local inflammatory reinforcement.

Beyond its direct effects on excitability, Piezo1 interacts functionally with other pain-related ion channels. Piezo1 activation has been shown to stimulate the opening of TRPV4, engaging phospholipase A2 activation and resulting in sustained intracellular Ca^2+^ elevation [44,45,46]. Although this mechanism has been described in endothelial cells and chondrocytes, a similar process may occur within nociceptive neurons, given that TRPV4 is transported distally along sensory nerves toward peripheral nerve endings, where it functions as an osmosensory transducer in primary afferent nociceptive fibres [47]. This extrapolation, however, provides indirect evidence inferred from other cell types, rather than direct observation within nociceptors themselves.

Piezo1 activation has furthermore been shown to potentiate the activity of members of the force-gated two-pore domain potassium K2P channel family, including TREK1, via conformational changes [14,48]. The underlying mechanism appears to involve tension-induced membrane alterations that shift Piezo1 from a curved to an expanded conformation [49], which opens the central pore. The process of pore opening requires displacement of the channel’s three propeller-like blades and produces deformation of the surrounding membrane bilayer that extends far beyond the boundaries of the protein itself [50]. Given that Piezo1 is a remarkably large transmembrane protein with a unique 38-transmembrane-helix topology and a total transmembrane thickness of approximately 29 Å [51,52], this conformation-based mechanism of potentiation may plausibly extend to other ion channels, including acid-sensing ion channels (ASICs), voltage-gated sodium channels, and members of the TRP channel family. Given that six TRP channels are expressed in primary afferent nociceptors and pain-sensing neurons [53], Piezo1-driven activation of this channel family may contribute meaningfully to pain hypersensitivity to thermal, chemical, and mechanical stimuli. These findings suggest that Piezo1 should be viewed not as an isolated mechanosensor, but as a constituent of a broader excitability network that can reshape nociceptor responses under pathological conditions. Interestingly, an acidic extracellular pH of ~6.5 attenuates human Piezo1-mediated currents by more than 80% and stabilises the channel in its inactivated state [54]. This protonation effect may be of particular relevance under pathological conditions, e.g., inflammation, in which tissue pH is dramatically reduced, since it would be expected to lower Piezo1 open probability during mechanical stimulation and thereby modulate neuronal excitability. Although this direct biophysical effect upon Piezo1 would be inhibitory in nature, a chronic acidic tissue environment can nonetheless upregulate Piezo1 expression in some non-neuronal cell types, indirectly sustaining local excitability.

Elevated PIEZO1 protein has been observed in DRG proximal to peripheral nerve injury [23], accompanied by greater Yoda-evoked Ca^2+^ elevation reported in peripheral sensory neurons and glial cells in models of peripheral nerve injury-induced neuropathy. Studies using mice with sensory neuron-specific Piezo1 deletion demonstrate that neuronal Piezo1 is required for dynamic light-touch mechanosensation and possibly for punctate mechanical force detection under physiological conditions [55]. These mice are also protected from acute and chronic tibial spared nerve injury-induced dynamic light-touch hypersensitivity, findings that together indicate a causal role for Piezo1 in mechanical hypersensitivity. At the same time, DRG neurons from uninjured Piezo1-deficient mice exhibit developmental compensation, including sensitised mechanically evoked currents and upregulation of Piezo2, TRPV1, and TRPV4 channels. This observation suggests that Piezo1 shapes mechanical responsiveness in peripheral nociceptors partly via compensatory channel networks. At peripheral nerve endings, Piezo1 acts as a mechanically activated nociceptive channel that relays pressure and tactile inputs to the DH of the spinal cord.

During inflammation, Piezo1 becomes sensitised and thereby contributes to mechanical allodynia, a condition in which normally innocuous touch is perceived as painful, contributing to a broader state of tactile hypersensitivity. Consistent with this role, silencing of Piezo1 in sensory pathways, in both mammalian neurons and in flies, reduces inflammatory and mechanical pain responses. Knockdown of Piezo1 in retinal ganglion cells has also revealed its importance for axon growth and regeneration [56], linking mechanotransduction not only to nociceptive hyperexcitability but also to structural plasticity.

Piezo1 activation in Schwann cells following peripheral nerve injury has been linked to elevated expression of senescence-associated genes that promote fibrotic scar formation surrounding peripheral nerves [57]. Piezo1 activation in both primary sensory neurons and peripheral glia associated with painful neuropathy therefore appears to reinforce the channel’s role in neuropathic pain and extends its influences beyond neurons alone, supporting an idea that peripheral nociceptive sensitisation arises from coordinated neuronal and glial responses to injury.

### 3.2. Functional Piezo1–TRPV1 Crosstalk

A key open question concerns how Piezo1 interacts with other nociceptor-enriched channels governing stimulus-specific sensitivity. In DRG neurons, Piezo1 is enriched in a subset of small-diameter DRG neurons co-expressing the TRPV1 marker [32]—TRPV1-positive nociceptors, also known as heat-sensitive, capsaicin-sensitive, or vanilloid receptor 1-expressing neurons. Piezo1 drives nociceptor hyperexcitability by converting mechanical stress into Ca^2+^ influx and membrane depolarisation, thereby increasing firing probability, and Piezo1 and TRPV1 appear to operate within a shared excitability network [30] rather than functioning as isolated channels. In TRPV1-positive nociceptors, Piezo1 knockdown reduces augmented TRPV1-mediated currents during inflammation and alters the excitability of TRPV1-expressing neurons; moreover, it alleviates mechanical allodynia in both acute (formalin model) and chronic (carrageenan-induced model) inflammatory pain [32]. This functional overlap is especially pertinent given that chronic pain rarely involves a single modality of sensitisation and instead reflects convergent mechanical, thermal, and inflammatory hypersensitivities.

The interaction between Piezo1 and TRPV1 in nociceptors appears to be bidirectional and activity-dependent. At the channel level, activation of TRPV1 by capsaicin can inhibit Piezo currents through Ca^2+^-dependent depletion of membrane phosphoinositides [58]. This mechanism engages activation of phospholipase Cδ and offers a plausible route by which thermal or chemical nociceptor activation may reshape Piezo-mediated mechanotransduction. Thus, inflammatory mediators and membrane depolarisation may sensitise Piezo1-mediated mechanotransduction, while TRPV1 activation can reciprocally reshape Piezo1-mediated currents through a similar Ca^2+^-dependent phosphoinositide pathway. Accordingly, Piezo1–TRPV1 crosstalk may constitute a mechanism through which distinct nociceptive modalities become functionally coupled. In pain states, this crosstalk between mechanotransduction and thermal pain signalling likely shifts the balance between mechanical and thermal sensitivity, helping to explain why inflammation or injury can further amplify mechanical hypersensitivity under pathological conditions. This bidirectional functional coupling supports a model in which Piezo1 helps drive mechanically evoked firing in nociceptors, while TRPV1 activation dampens Piezo-mediated mechanotransduction by altering the local membrane lipid environment.

Taken together, these findings suggest that Piezo1 may form a mechanistic bridge between mechanical force detection and multimodal nociceptor hyperexcitability—a relationship that strengthens the view that peripheral sensitisation arises from coordinated channel activity (or even synergy) rather than from the action of any single receptor in isolation.

### 3.3. Piezo1 at the Neuro-Skeletal Interactions

Piezo1 may also influence nociception indirectly through its involvement in musculoskeletal tissues located adjacent to the spinal axis, including osteoblasts, osteocytes, cartilage, and intervertebral disc cells. Piezo1 is expressed in inflammatory cells, such as neutrophils, macrophages and endothelial cells, as well as non-inflammatory cells, including osteoblasts, osteoclasts, periodontal cells, and chondrocytes [45,59,60], contributing to tissue mechanobiology and homeostasis [61]. Aberrant Piezo1 signalling has been linked to disrupted osteoblast–osteoclast coupling, cartilage degeneration, and disc cell senescence, processes that collectively alter both the mechanical and inflammatory environment surrounding dorsal roots and spinal ganglia (for review, see [62,63,64]). In this context, dysregulated Piezo1 activity within both neural and stromal compartments may act synergistically to increase peripheral nociceptive input and sustain hypersensitivity at the spinal cord level. Consistent with this, administration of the Piezo1 agonist Yoda1 worsens pain-related phenotypes, for example by exacerbating osteoarthritis-associated mechanical hyperalgesia [65], whereas the Piezo1 blocker Grammostola spatulata mechanotoxin-4 (GsMTx4), when combined with exercise, improves skeletal muscle structure and motor function following spinal cord injury (SCI) [66]. These findings indicate that abnormal Piezo1 activation contributes to pain signalling under altered mechanical conditions following injury, such as increased tissue stiffness. This broader tissue perspective is important because persistent nociceptive drive is shaped not only by neurons, but also by the mechanical and inflammatory microenvironment that surrounds them.

Within musculoskeletal structures, Piezo1 activation enhances the differentiation of skeletal muscle precursors [67] and promotes muscle regeneration [68]. Mechanosensitive Piezo1 channel also plays a key role in bone formation and cartilage homeostasis [43,69]. Conditional Piezo1 knockout increases bone formation [70], and elevated Piezo1 expression in osteoblasts and osteocytes helps coordinate load-dependent adaptation [71,72]. When Piezo1 signalling becomes dysregulated, or Piezo1 is inactive, osteoblast–osteoclast crosstalk is disrupted [60], cartilage breakdown accelerates, and intervertebral disc cells undergo senescence, ultimately contributing to spinal degenerative disease. Because mechanical changes affect the local microenvironment surrounding spinal ganglia and dorsal roots, dysregulated Piezo1 activity in both neural and stromal compartments may act synergistically to drive persistent nociceptive input and nociplastic hypersensitivity within the DH.

Piezo1 may link tissue degeneration to sustained nociceptor activation by integrating local mechanical stress with inflammatory signalling. The pro-inflammatory mediator IL-1α has been shown to upregulate Piezo1 expression in porcine chondrocytes and to increase intracellular Ca^2+^ levels, accompanied by Piezo1 channel opening during chondrocyte responses to inflammation and mechanical trauma in osteoarthritis [43]. These changes reshape the local microenvironment of spinal ganglia and dorsal roots, favouring ongoing nociceptor activation. Thus, Piezo1 has a position at the intersection of neuro- and osteo-mechanobiology, acting as a multifaceted contributor to painful hypersensitivity.

Overall, the available experimental evidence positions Piezo1 as a multifaceted channel, acting through cell-specific and context-dependent mechanisms across the nociceptive axis.

## 4. Piezo1 Mechanotransduction in Central Sensitisation

Mechanical or inflammatory activation of peripheral Piezo1 associated with Ca^2+^ influx enhances synaptic transmission, strengthening nociceptive drive onto DH neurons. In the context of chronic pain, Piezo1-mediated signalling in spinal nociceptive neurons may interact with inflammatory mediators released by activated glia, resulting in sustained circuit activation and hypersensitivity (Figure 1). Piezo1-driven mechanotransduction may therefore contribute to a hypothetical shift from peripheral to central sensitisation, representing a critical transition from acute to chronic pain [73]. In this way, Piezo1 may act as a potential synergistic contributor to nociplastic hypersensitivity, particularly where altered mechanotransduction intersects with the neuroinflammatory sensitisation of central circuits associated with chronic pain states.

### 4.1. Piezo1 and Synaptic Potentiation in Nociceptive Circuits

Following nerve injury, such as in spared nerve injury models, Piezo1 undergoes rapid axonal transport to both peripheral nerve endings and the central terminals that innervate the DH of the spinal cord [23,74]. Once peripheral input reaches the DH terminals, Piezo1 may contribute to central sensitisation, defined as the activity-dependent amplification of nociceptive transmission within central circuits, thereby proving a mechanistical link between peripheral sensitisation and central circuit plasticity via Piezo1 activation along the primary afferent pathway.

One mechanism through which Piezo1 may promote pathological cell signalling within central circuits is through altered membrane composition and, consequently, altered channel kinetics. In N2A cells, reduction in membrane cholesterol largely slows the inactivation kinetics of Piezo1 channels [75], an effect that would prolong channel opening following activation. Because inflammatory conditions are associated with lowered cholesterol content in sensory DRG neurons [76], this mechanism could provide a basis for prolonged Piezo1 activation during nociceptive transmission and, consequently, for neuronal hyperexcitability. A similar mechanism has been described for the voltage-gated Nav1.9 channel, wherein membrane cholesterol depletion promotes both mechanical and thermal hyperalgesia [76].

The other potential mechanism concerns the negative impact of Piezo1 on axon myelination. Pharmacological activation of Piezo1 has been associated with CNS demyelination, demonstrated within the cerebral cortex [77], an effect that may promote pathological signalling by altering membrane mechanics, impairing conduction fidelity, and increasing axonal susceptibility to ectopic firing. Conversely, Piezo1 inhibition has been shown to reduce demyelination after intracerebral haemorrhage [78]. On the basis of this indirect evidence, changes in Piezo1 kinetics may prolong nociceptive input and drive spinal neuronal hyperexcitability during painful states (Figure 1).

Changes in Piezo1 expression and function have also been linked to augmented circuit excitability within central pain pathways. Increased PIEZO1 protein levels have been observed in inhibitory parvalbumin-positive interneurons within the anterior cingulate cortex following spared sciatic nerve injury, but not in glutamatergic CaMKII-positive neurons [20]. This upregulation involves activation of the NLRP3 inflammasome and downstream Ca^2+^/NF-κB signalling, leading to the production of inflammatory cytokines [79]. An abnormal Piezo1 level in inhibitory interneurons causes Ca^2+^ excitotoxicity and cellular damage, which in turn initiates inflammasome-driven microglial phagocytosis of these interneurons. The resulting loss of inhibitory tone disrupts the excitation–inhibition (E/I) balance within the anterior cingulate cortex and exacerbates neuronal hyperexcitability. Although these findings derive from supraspinal circuitry, they remain conceptually relevant since they illustrate how Piezo1-dependent loss of inhibition could contribute to sensitisation within spinal networks. Within the DH of the spinal cord, E/I imbalance is a key mechanism of central sensitisation [73,80,81] and would be expected to facilitate the abnormal transmission of nociceptive signals to higher pain centres.

Piezo1 may further contribute to synaptic dysfunction and neurodegeneration through Ca^2+^-dependent mechanisms of cytotoxicity. Sustained Piezo1 activation can elevate intracellular Ca^2+^ levels in neurons, leading to overactivation of calcium/calmodulin-dependent protein kinase IIα (CaMK2α). This, in turn, promotes oxidative stress and apoptosis via the p-CaMK2α/ERK/CREB and ox-CaMK2α/MAPK p38/NFκB p65 pathways, ultimately impairing synaptic function and cognitive impairment in mice [82]. Supporting a role for Piezo1 in synaptic function, the Piezo1-mediated Calpain-1/ERK pathway has also been implicated in hippocampal synaptogenesis and post-surgical memory defects in aged mice [83]. Although evidence remains indirect to nociceptive pathways, it nonetheless reinforces the broader idea that sustained Piezo1 activity can alter synaptic function beyond immediate membrane depolarisation alone.

Structurally, the considerably large size of Piezo1 channels likely precludes their localisation within the postsynaptic density at the synapses. Instead, the molecular environment surrounding Piezo1 at the cell surface comprises a PIEZO1-proximal interactome enriched in surface proteins localised to cell junctions and signalling hubs within the plasma membrane, and Piezo1 is additionally bound intracellularly to a Myo-D-family inhibitor transcriptional regulator that serves as an auxiliary channel subunit [84]. The identified interaction partners at the cell surface include CADM1 and GPC4 proteins, together with the adhesion molecule CADM1/SynCAM, which slows Piezo1 inactivation kinetics with little effect on Piezo2 [85]. CADM1, an adhesion molecule, and SynCAM proteins are recognised contributors to synapse organisation and function, with SynCAM1 and SynCAM2 proteins expressed in neurons at both excitatory and inhibitory synapses [86,87]. These interactions suggest that Piezo1 may influence synaptic signalling through microdomains and adhesion-dependent regulatory complexes notwithstanding its apparent exclusion from the postsynaptic density.

Piezo1 is likewise expressed on nanoscale astrocytic processes, enabling astrocytes to respond directly to mechanical stimulation through the generation of cationic currents, Ca^2+^ influx, and ATP release [21]. This mechanosensitivity of astrocytes complements the well-established role in regulating synaptic function, plasticity, and circuit activity [88,89]. Piezo1 activation in astrocytes enhances NMDA receptor-dependent currents, thereby influencing astrocyte–neuron interaction and modulating neuronal excitability [90]. Astrocyte-specific Piezo1 knockout mice exhibit impaired synaptic potentiation (i.e., deficient hippocampal LTP), accompanied by decreased adult neurogenesis and impairments in learning and memory. Given that DH sensitisation is thought to represent a specific form of spinal plasticity that underlies pain of various origins, which is primarily mediated by glutamate receptor dysfunction [91], Piezo1-mediated hyperexcitation may likewise contribute to maladaptive spinal plasticity in the DH.

Although these findings derive predominantly from CNS regions beyond the spinal DH rather than providing direct evidence from spinal circuits, they nonetheless collectively point toward potential roles for Piezo1 in neuronal and glial signalling, synaptic organisation, and E/I balance that may converge within the spinal DH to enhance synaptic efficacy and drive maladaptive synaptic potentiation, hence central sensitisation.

### 4.2. Piezo1 Activation Within the DH: Plausible Triggers

Piezo1 is gated principally by lateral membrane tension: increased tension flattens the channel’s characteristically curved, dome-shaped conformation, precipitating outward flexion of its three propeller-like blades and consequent opening of the central pore [49,50,51,52]. In principle, several distinct sources of mechanical force could plausibly generate such tension within DH tissue during chronic pain states.

Cell swelling associated with neuroinflammation or oedema, together with the increased tissue stiffness that accompanies gliosis, scarring, or extracellular matrix remodelling following injury, could raise local membrane tension to a degree sufficient to activate Piezo1 [92,93,94]. Vascular forces represent a further plausible mechanism, taking into account that Piezo1 in endothelial and vascular smooth muscle cells is a well-established sensor of shear stress and intravascular pressure, where fluid flow-induced deformation activates the channel to regulate vascular tone and structure [26,27]. Haemodynamic forces acting on the spinal microvasculature could extend to perivascular glia and neurons during prolonged neuroinflammation or altered regional blood flow. Piezo1-dependent astrocytic and microglial remodelling may additionally alter the local mechanical microenvironment, recruiting neuronal Piezo1. To date, only membrane tension is directly supported by biophysical evidence specific to Piezo1 [49,50,51,52], whereas cell swelling, tissue stiffness, vascular forces, and glial remodelling remain plausible, yet largely untested, mechanisms within the DH that should be examined in future studies.

### 4.3. Piezo1-Mediated Neuroinflammatory Mechanisms

Piezo1 activation has been shown to modulate the release of cytokines and chemokines, specifically TNF-α, IL-1β, and IL-6, by initiating intracellular Ca^2+^ signalling and inhibiting the NF-κB inflammatory pathway [95,96], thereby linking mechanotransduction to neuroinflammatory signalling. Studies of brain and spinal cord pathology have reported Piezo1-mediated Ca^2+^ transients and cytokine release in glial cells exposed to mechanical stress, indicating that Piezo1 contributes to neuroinflammatory signalling within the DH.

Following SCI or trauma, Piezo1 is upregulated in resident microglia and astrocytes proximal to the lesion, where it modulates ferroptosis-related ion dysregulation and inflammatory activation [41]. Microglia, the primary resident immune cells of the CNS, influence circuit function through monitoring, phagocytosis, and synaptic pruning and participate in both injury and repair responses (for review, [97,98]). Following SCI, the mechanical properties of spinal tissue undergo dramatic changes, with cell swelling, local matrix remodelling, increasing tissue stiffness and pronouncing Piezo1 activation. During the early stages of injury, Piezo1 is markedly upregulated in microglia and macrophages, and Piezo1-mediated Ca^2+^ influx has additionally been shown to activate the C1q–C3 complement pathway [99,100]. This drives a pro-inflammatory response that can exacerbate secondary tissue damage, creating a feedback loop that sustains hyperexcitation of spinal nociceptive neurons and contributes to maladaptive DH circuit changes associated with E/I imbalance and central sensitisation. Consistent with this, inhibition of Piezo1 in immune cells significantly reduces inflammation and promotes recovery of motor function in mice. In nucleus pulposus cells of the intervertebral discs, Piezo1 activation promotes NLRP3 inflammasome assembly, and cumulative evidence indicates that the NLRP3 inflammasome is likewise assembled within microglia in chronic neuropathic pain [101].

Inflammatory mediators can reciprocally activate Piezo1. Cytokines associated with neuroinflammation, as well as lipopolysaccharide, an inflammatory stimulator, have been reported to activate Piezo1 [43], and TNF-α has been shown to induce Piezo1 upregulation in human astrocytes [22]. Activated Piezo1 triggers Ca^2+^ influx and can additionally promote Ca^2+^ release from intracellular stores [26,27,28]. Disturbance of the immune microenvironment within the DH during inflammation, injury, or trauma may therefore activate Piezo1 and contribute to circuitry hyperexcitability. Astrocyte-specific Piezo1 deletion causes a striking reduction in hippocampal tissue volume and brain weight and severely impairs neurogenesis [21]. Conversely, the Piezo1 inhibitor GsMTx4 exerts neuroprotective effects by blocking chemically induced astrocyte toxicity and microglial activation, thereby supporting a pro-inflammatory role for Piezo1 in certain contexts. At the same time, however, Piezo1-mediated Ca^2+^ signalling has been reported to inhibit LPS-induced NF-κB signalling and cytokine production within microglia [96], while the PIEZO-Ca^2+^-CREB axis has been identified as a pivotal driver of glial activation that orchestrates mitochondrial fusion and neurotrophic support [102]. These seemingly divergent findings indicate that Piezo1 signalling in glia may be either pro-inflammatory or protective, depending on cell type, disease stage, and the nature of stimulus involved (e.g., mechanical or chemical).

Piezo1 also exerts influence over oligodendrocyte-lineage cells and myelination. Piezo1 activation has been reported to inhibit oligodendrocyte proliferation and migration within the CNS [103]. Inhibition of Piezo1 with GsMTx4 slows demyelination, whereas activation with Yoda1 can damage central axons, as observed, for example, in Purkinje neurons [77]. Inhibition of Piezo1 also reduces endoplasmic reticulum stress and cellular apoptosis and protects the myelin sheath, thereby improving remyelination and the fidelity of neuronal signal transfer after injury [78]. These findings broaden the scope of Piezo1-driven impact beyond neuron–glia signalling alone to encompass myelin integrity and axonal conduction, both of which may shape nociceptive circuit output.

In astrocytes, Piezo1 signalling promotes morphological changes and increases cellular stiffness, which directly contributes to the formation of a dense glial scar [104]. Because scar tissue can physically impede axon regrowth and tissue regeneration, Piezo1 targeting has been proposed as a strategy to reduce scar rigidity and facilitate wound repair [105], an effect that involves cytoskeletal reorganisation via activation of the Wnt7b-Ca^2+^-dependent nonclassical signalling pathway [106].

Taken together, these findings indicate that Piezo1 integrates mechanical and inflammatory signalling in glial cells. Depending on the cellular context and disease stage, Piezo1 may either amplify neuroinflammation, complement activation, and maladaptive plasticity or alternatively engage protective glial programmes. Within the DH, the balance of current evidence points toward a predominantly pro-inflammatory, pro-sensitising role for Piezo1 in the initiation and maintenance of nociplastic hypersensitivity; however, the whole picture is still not fully understood in the DH and requires further studies.

## 5. Therapeutic Targeting of Piezo1 in Pain and Spinal Pathology

Piezo1 is essential for normal mammalian development and physiology, which immediately highlights its therapeutic promise simultaneously with the risk of systemic adverse effects associated with Piezo1 modulation. Global deletion of *Piezo1* causes embryonic or perinatal lethality in mice [107], and endothelial-specific *Piezo1* knockout is similarly embryonic lethal [26]. Because Piezo1 is widely expressed throughout the body and contributes to fundamental physiological processes, including the regulation of blood pressure and red blood cell volume, it presents an inherently challenging therapeutic target, and any strategy directed at Piezo1 must carefully balance efficacy against tissue selectivity and safety.

Despite these concerns, Piezo1 has emerged as an attractive target in inflammatory and neuropathic pain, as well as in tissue repair. Pharmacological inhibition with GsMTx4, when applied continuously to the sciatic nerve, reduces mechanical hypersensitivity after nerve injury [23], and Piezo1 knockdown alleviates tactile hypersensitivity in inflammatory pain models [32]. Gene-targeting approaches via conditional loss of *Piezo1* similarly reduce responses to dynamic mechanical stimulation of the hind paw [55]. In peripheral tissues, deletion of Piezo1 from epidermal keratinocytes attenuates chemotherapy-induced mechanical hypersensitivity in mice [108], further supporting the idea that targeting Piezo1 could help normalise somatosensory pain processing.

Piezo1 targeting may also benefit neural repair. Deletion of Piezo1 in macrophages and microglia ameliorates inflammation and improves functional recovery following SCI [41]. Targeting Piezo1 in astrocytes has been proposed to reduce tissue scar rigidity, given that a dense scar structure physically impedes axon regrowth and tissue regeneration; by modulating cellular stiffness, this strategy may facilitate wound repair [105]. Piezo1 targeting therefore has dual therapeutic relevance, modulating both nociceptive signalling and the structural environment for recovery after tissue injury.

### 5.1. Drug Screening for Highly Selective Piezo1 Modulators

At present, the most clinically relevant approach remains pharmacological modulation. However, systemically administered Piezo1 blockers would likely produce severe side effects, including vascular dysfunction and anaemia, thereby impeding clinical translation because of off-target toxicity. For this reason, current efforts are directed toward drug screening to identify more selective inhibitors and safer delivery strategies, with particular emphasis on local rather than systemic administration.

Several inhibitors are presently under investigation as candidate Piezo1-targeting compounds. GsMTx4, a tarantula venom-derived peptide is the best-characterised inhibitor of mechanosensitive channels and remains the most widely used experimental tool, although it also targets Piezo2 and TRPV4 [109,110]. GsMTx4 has been evaluated in preclinical models of ischaemia–reperfusion injury [111], where it reduced ischaemic inflammation, reactive oxygen species generation, and calcium dysregulation. Local peripheral administration of GsMTx4 has also been explored in tumour-related pain, including schwannoma-associated pain, reducing mechanical hypersensitivity [112] and preserving muscle mass with decreased susceptibility to contraction-induced injury [113]. Given that GsMTx4 lack selectivity for Piezo1, these effects provide rather indirect evidence for a Piezo1-specific role in persistent pain. A further commonly used compound is Dooku1, a small-molecule synthetic antagonist designed to block Yoda1-induced Piezo1 activation by competing with the agonist; it does not, however, function as a general blocker of mechanically activated Piezo1. Although Dooku1 is a valuable structural template for drug development, its poor aqueous solubility limits its clinical applicability.

There is ongoing screening of existing drugs and natural compounds for Piezo1-specific activity, ideally for FDA-approved drugs. Among those, benzbromarone, a drug used in the treatment of gout, has recently been identified as a Piezo1 inhibitor [114], although its analgesic effects and safety profile remain to be established. Several natural compounds, such as Tubeimoside I, escin, and jatrorrhizine, have also demonstrated inhibitory effects on Yoda1-evoked Piezo1 activation [115] and have been linked to reduced vascular inflammation in endothelial cells [116] or to attenuation of mechanical stretch-induced inflammatory responses via the Piezo1-mediated NF-κB pathway [117]. Notably, a GsMTx4-based short peptide reduced mechanical hyperalgesia and neuropathic pain without affecting motor behaviours or thermal-induced pain, acting through TRPV4 rather than through Piezo1 only [118]. Altogether, direct and indirect evidence supports Piezo1 inhibition as a promising strategy for modulating inflammatory signalling, neuroinflammation, and pain, while simultaneously underscoring the need for more selective pharmacological tools.

Several important gaps remain before Piezo1 can be considered a validated therapeutic target. First, the majority of available evidence comes from preclinical models, so human relevance, optimal dosing, and long-term safety are still unknown. Second, many available inhibitors lack high selectivity for Piezo1 and may additionally affect other channels, complicating interpretation and increasing the risk of off-target effects. Third, the physiological functions of Piezo1 in vasculature, erythrocytes, and other tissues raise legitimate concern that systemic inhibition could produce toxicity, making tissue-targeted delivery essential. Finally, it remains unclear whether all pain modalities would benefit from Piezo1 targeting or whether the greatest therapeutic effect will emerge from combined targeting of mechanotransduction networks.

### 5.2. Delivery Strategies for Piezo1 Targeting in the CNS

A major limitation confronting Piezo1 pharmacology is the lack of delivery strategies capable of achieving sufficient exposure within CNS circuits without perturbing physiological functions elsewhere across multiple organ systems. Systemic administration of Piezo1 inhibitors carries a high risk of vascular, hematologic, and organ-level side effects. Most Piezo1 modulators remain incompletely characterised with respect to their pharmacokinetics, tissue distribution and long-term safety—small molecules often exhibit poor solubility and unfavourable absorption, distribution, and excretion profiles, whereas peptide-based inhibitors (e.g., GsMTx4) are prone to rapid proteolysis and renal clearance. The synthesised D-enantiomer, GsMTx4-D, resists enzymatic breakdown and exhibits a longer tissue half-life [111]. For spinal applications specifically, the blood–spinal cord barrier further limits the feasibility of systemic delivery, often necessitating direct intrathecal injections or the development of engineered delivery systems. Although local administration can reduce systemic exposure while achieving high local concentrations, this approach is invasive, technically demanding, and may itself induce tissue injury or inflammation. Moreover, local delivery does not address the issue of cell-type specificity, considering that Piezo1 is expressed in neurons, astrocytes, microglia, oligodendrocyte-lineage cells, and vascular components.

To overcome these limitations, nanomaterial-based carriers and ligand-conjugated prodrugs offer potential routes for delivering Piezo1 modulators to sites of injury while minimising systemic exposure. Chitosan-stabilised bovine serum albumin nanoparticles loaded with GsMTx4 improved outcomes in lung injury and inhibited cellular apoptosis [119], while mannose-modified liposomes targeting synovial macrophages alleviated osteoarthritis progression [120]. For CNS targeting, promising approaches should consider blood–brain barrier-penetrating nanoparticles and intrathecal or intracerebroventricular delivery combined with controlled-release formulations. In parallel, gene-based strategies, such as RNA interference, antisense oligonucleotides, or CRISPR-based modulation delivered via viral vectors, may enable cell-type-specific manipulation of Piezo1, particularly when combined with cell-specific promoters or tropism-engineered vectors. These approaches can, in principle, provide spatial and temporal precision that would otherwise be difficult to achieve, though they also raise concerns regarding immunogenicity, off-target effects, and long-term safety.

## 6. Conclusions

To summarise, Piezo1 is a biologically promising yet therapeutically challenging target. Preclinical studies, encompassing both direct and indirect evidence, support a role for Piezo1 in mechanical hypersensitivity, inflammatory and neuropathic pain, and glial scar remodelling, making it a promising candidate for both analgesia and tissue repair. However, its essential physiological functions create a narrow therapeutic window for precision targeting, and the field currently lacks highly selective, clinically suitable Piezo1 inhibitors. Progress in Piezo1-targeted therapeutics will therefore depend on the co-optimisation of pharmacology and delivery, including (i) the design of inhibitors with improved solubility, stability, and CNS penetration, (ii) the development of tissue- and cell-type-specific delivery platforms, including nanoparticles, prodrugs, and viral vectors, and (iii) the strategic exploitation of local or controlled-release administration as appropriate.

## Data Availability

No new data were created or analysed in this study. Data sharing is not applicable to this article.
